# Diagonal earlobe crease and long-term survival after myocardial infarction

**DOI:** 10.1186/s12872-021-02425-4

**Published:** 2021-12-16

**Authors:** Christian Thilo, Christine Meisinger, Margit Heier, Wolfgang von Scheidt, Inge Kirchberger

**Affiliations:** 1 Medizinische Klinik, Romed Klinikum Rosenheim, Rosenheim, Germany; 2 Chair of Epidemiology, University Augsburg, University Hospital Augsburg, Stenglinstr. 2, 86156 Augsburg, Germany; 3 Independent Research Group Clinical Epidemiology (KEPI), Helmholtz Zentrum München, German Research Center for Environmental Health (GmbH), Neuherberg, Germany; 4 Institute of Epidemiology, Helmholtz Zentrum München, German Research Center for Environmental Health (GmbH), Neuherberg, Germany; 5 KORA-Study Centre, University Hospital Augsburg, Augsburg, Germany; 6 Department of Cardiology, University Hospital Augsburg, Augsburg, Germany; 7 Centro de Investigación Biomédica en Red, Enfermedades Cardiovasculares (CIBERcv), Madrid, Spain

**Keywords:** Myocardial infarction, Diagonal earlobe crease, Frank’s sign, Survival

## Abstract

**Background:**

The association between the presence of a diagonal earlobe crease (DEC) and coronary artery disease has been prescribed earlier. However, it is unclear whether patients with acute myocardial infarction (AMI) and DEC have a higher risk of dying.

**Methods:**

Study participants were persons with AMI who were included in the KORA Myocardial Infarction Registry Augsburg from August 2015 to December 2016. After taking pictures of both earlobes, two employees independently assessed the severity of DEC in 4°. For analysis, the expression of the DEC was dichotomized. Information on risk factors, severity and therapy of the AMI was collected by interview and from the medical record. Vital status post AMI was obtained by population registries in 2019. The relationship between DEC and survival time was determined using Cox proportional hazards models.

**Results:**

Out of 655 participants, 442 (67.5%) showed DEC grade 2/3 and 213 (32.5%) DEC grade 0/1. Median observation period was 3.06 years (5–1577 days). During this period, 26 patients (12.2%) with DEC grade 0/1 and 92 patients (20.8%) with grade 2/3 died (hazard ratio 1.91, 95% confidence interval (CI) 1.23–2.96, *p* = 0.0037). In the fully adjusted model, patients with DEC grade 2/3 had a 1.48-fold increased risk of death compared to the DEC grade 0/1 patient group (CI 0.94–2.34, *p* = 0.0897). The fully adjusted model applied for 1-year survival revealed a significant, 2.57-fold hazard ratio of death (CI 1.07–6.17, *p* = 0.0347) for the patients with DEC grade 2/3.

**Conclusions:**

Our results indicate that DEC is independently associated with 1-year AMI survival.

**Supplementary Information:**

The online version contains supplementary material available at 10.1186/s12872-021-02425-4.

## Background

**Fig. 1:**
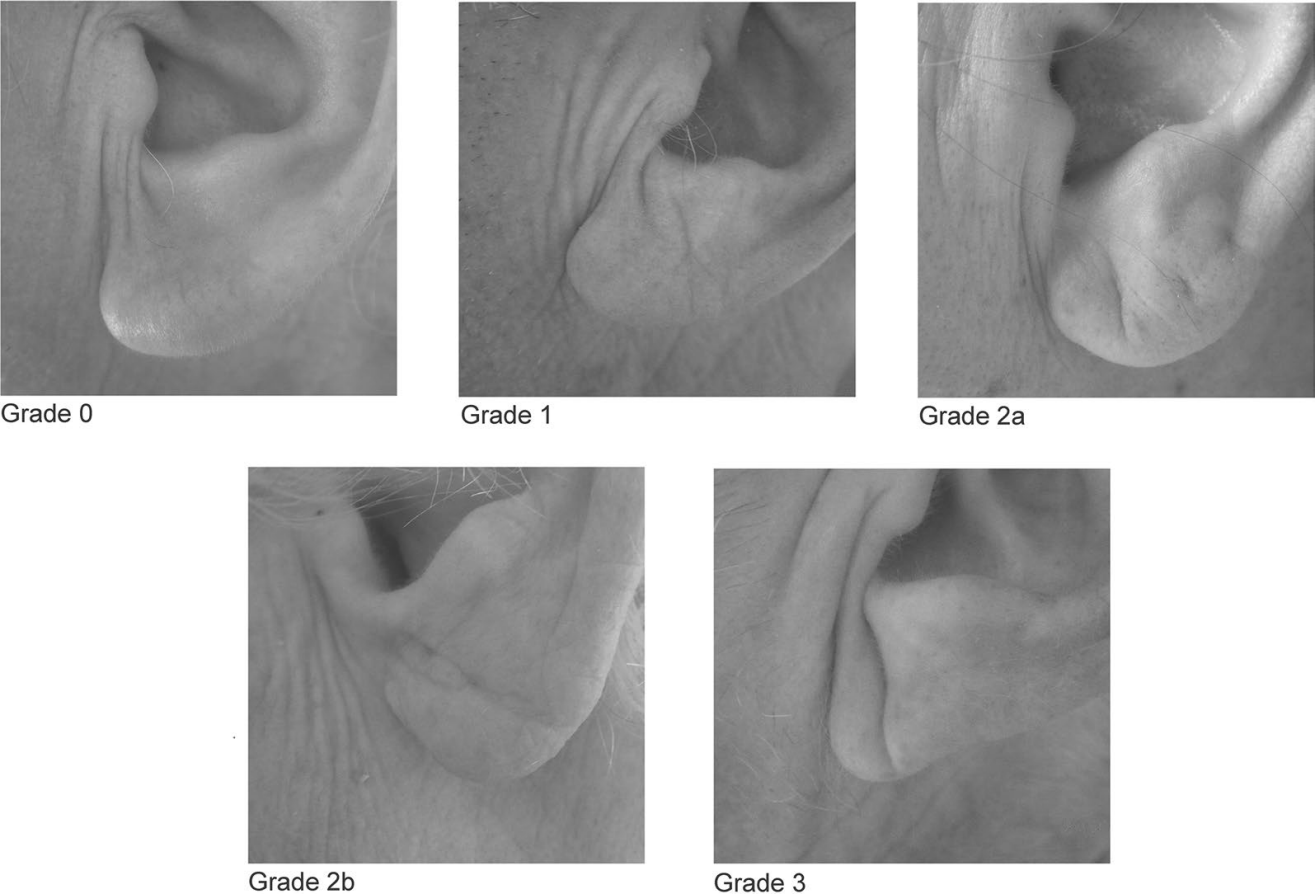
Examples of ear lobe crease classification

Coronary artery disease (CAD) is the most common disease in Europe and in the US. In the US, CAD accounts for 1 in 7 deaths, killing over 366,800 people a year [1, 2]. For decades, factors that promote CAD have been investigated. In addition to classic risk factors, the diagonal earlobe crease (DEC) has been described as an independent indicator for CAD and other vascular diseases [3]. DEC was first introduced in 1973 by S.T. Frank and is subsequently also described as “Frank's sign” [4]. Frank [4] defined DEC as a deep prominent crease in the lobule portion of the auricle. In order to distinguish between different manifestations of DEC, the classification by Patel [5] considered extent and deepness of the crease resulting in 5 grades (see Fig. 1).

The pathogenesis of Frank's sign is based on several theories. One concept considers primarily a microvascular disorder in the earlobe with elastic fiber tears and thickening of the arteries [6]. Others reported shortened telomeres in patients with metabolic syndrome that may indicate atherosclerosis driven accelerated aging [7, 8]. Recently, a context of (bilateral) Frank's sign with vascular dysfunction has been identified [9]. Another hypothesis discusses collagen degeneration as a cause of atherosclerosis formation also occurring in the skin [5]. In autopsy, risk of death from acute myocardial infarction (AMI) was doubled with at least moderate two-sided DEC [5].

Although previous studies have shown that DEC is an independent indicator of CAD, its association with the prognosis after AMI is not investigated so far. Thus, the objective of the present study was to prospectively examine the association of Frank's sign on long-term survival in patients with AMI.

## Methods

The study was conducted in accordance to the Declaration of Helsinki and approved by the ethics commission of the Bavarian Medical Association (Approval No. 15016, 28/04/2015). From all participants written informed consent was obtained.

**Fig. 2:**
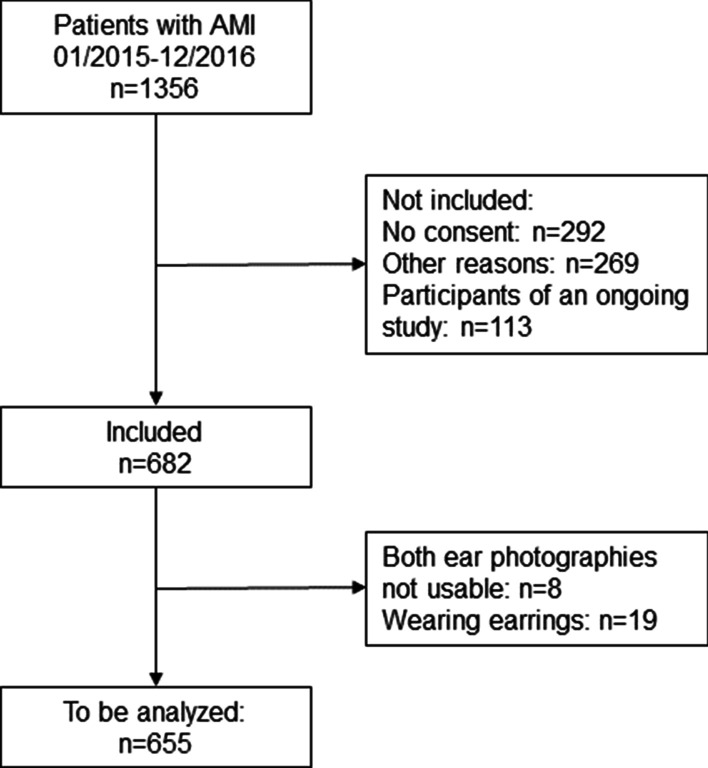
Flow diagram

The study population consisted of patients with AMI who were admitted to a hospital in the study region of the KORA Myocardial Infarction Registry. Figure 2 shows flow diagram of eligibility. The population-based AMI registry was implemented in 1984 as part of the WHO-MONICA (Monitoring Trends and Determinants in Cardiovascular Disease) project. After the termination of MONICA in 1995, the registry became part of the framework of KORA (Cooperative Health Research in the Region of Augsburg). Since 1984, all cases of coronary deaths and non-fatal AMI of the 25–74 year old study population in the city of Augsburg and the two adjacent counties (about 600,000 inhabitants) have been continuously registered [10, 11]. In 2009, the age range of participants was extended to 84 years.

In the present study, patients who were registered at the KORA Myocardial Infarction Registry between 1 August 2015 and 31 December 2016 were contacted by a study nurse during their hospital stay after transfer from the intensive care unit. They received information about the study and were asked to participate.

Photos of both ear lobes were taken in sitting position by a study nurse. All photos were rated for the presence of a DEC by two trained study nurses independently. The raters were blind towards the rated patients and their clinical characteristics. The results of the two raters were compared and in case of disagreement an third person discussed the findings with the raters and a common decision was made. The assessment was based on the classification published by Patel et al. [9]: grade 0 is no crease, grade 1 is any degree of crease less than 2, grade 2a is a deep diagonal crease greater than 50% but less than 100% across the lobe, grade 2b is a complete crease across the lobe that is superficial but not deep, grade 3 is a deep crease across the whole lobe (see Fig. 1).

From the KORA Myocardial Infarction Registry, socio-demographic and clinical data collected by patient interview and chart review was used. Clinical information which was not routinely collected for the KORA Myocardial Infarction Registry, was extracted from the medical charts. Information on current vital status and date of death was obtained from the population registries in 2019.

Continuous data were expressed as median values with interquartile ranges (IQR) or means with standard deviation and categorical variables as percentages. The Chi^2^-test was used to test the differences in frequencies, t-Test or Wilcoxon test for differences in continuous variables, respectively.

Agreement between the raters was determined by weighted Kappa coefficients and related 95% confidence intervals.

To investigate the association between DEC and mortality, relative risks were computed by Cox proportional hazards models. The proportional hazards assumption was valid for all factors used in the Cox models shown by parallel lines of log(− log(event)) versus log of event times. An unadjusted model, a model adjusted for age and sex as well as a model adjusted for all meaningful potential confounding variables was calculated. Potential confounding factors were sex (male/female), age (continuous), history of reinfarction (yes/no), history of diabetes mellitus (yes/no), history of stroke (yes/no), chronic kidney disease (yes/no), peripheral arterial occlusive disease (yes/no), any recanalization therapy (yes/no), number of affected vessels (1/2/3/others), c-reactive protein level (≤ 3 vs. > 3 mg/dl), left ventricular ejection fraction < 50% versus ≥ 50%. Variables with a *p* value < 0.05 were taken to be statistically significant.

For the main analysis, the DEC variable was dichotomized by collapsing grades 0 and 1, and grades 2a, 2b and 3. The ear with the highest grade was considered. Sensitivity analyses were performed with different combinations of gradings: grades 0, 1, 2 (2a and 2b) and 3, sum of grades of both ears, grad 3 versus grades 2–0.

## Results

We included 655 patients with AMI in our study (mean age 68.0 ± 10.8 years) (see Fig. 1). The weighted kappa coefficient of agreement for DEC classification was 0.68 (95% confidence interval (CI) 0.64–0.71). Median observation period was 1117 days (5–1577 days). Further characteristics are detailed in Table 1 (Angiographic findings are presented in Additional file 1).

**Table 1:** Demographic and clinical characteristics of the study population with and without diagonal earlobe crease (n = 655)

	Total		DEC grade 0/1		DEC grade 2/3		*p* value
	Mean	SD	Mean	SD	Mean	SD	
Age	67.95	10.78	62.43	11.60	70.60	9.26	< 0.0001
C-reactive protein	1.85	8.88	1.81	4.46	1.87	5.27	0.0287

	n	%	n	%	n	%	*p* value
*Gender*							
Male	490	74.81	180	84.51	310	70.14	< 0.0001
Female	165	25.19	33	15.49	132	29.86	
*Risk factors*							
Smoking							
Current smoker	181	27.72	80	37.74	101	22.90	0.0003
Ex smoker	241	36.91	65	30.66	176	39.91	
Never smoker	231	35.38	67	31.60	164	37.19	
Hypertension	529	80.76	159	74.65	370	83.71	0.0058
Diabetes	233	35.57	75	35.21	158	35.75	0.8934
Hyperlipidemia	428	65.34	127	59.62	301	68.10	0.0327
Coronary heart disease	194	29.62	50	23.47	144	32.58	0.0168
Heart failure	73	11.15	16	7.51	57	12.90	0.0402
Renal failure	90	13.74	14	6.57	76	17.19	0.0002
COPD	47	7.18	14	6.57	33	7.47	0.6782
Angina pectoris	90	13.74	22	10.33	68	15.38	0.7830
PAOD	85	13.00	21	9.91	64	14.48	0.1035
Stroke	67	10.23	12	8.92	48	10.86	0.4429
Obesity (BMI > 30)	183	27.94	57	26.76	126	28.51	0.6408
Xantelasmata							
Yes	18	2.75	4	1.89	14	3.17	0.3762
No	625	95.57	206	97.17	419	94.80	
Unclear	11	1.68	2	0.94	9	2.04	
*AMI characteristics*							
Reinfarction	148	22.60	41	19.25	107	24.21	0.1551
STEMI	229	34.96	84	39.44	145	32.81	0.0049
NSTEMI	331	50.53	113	53.05	218	49.32	
Bundle branch block	69	10.53	12	5.63	57	12.90	
Not determined	26	3.97	4	1.88	22	4.98	
1-vessel disease	167	26.13	59	27.96	108	25.23	0.1543
2-vessel disease	190	29.73	71	33.65	119	27.80	
3-vessel disease	265	41.47	76	36.02	189	44.16	
Main artery disease	14	2.19	3	1.42	11	2.57	
Diffuse stenosis or absence of coronary artery disease	3	0.47	2	0.95	1	0.23	
*AMI treatment and outcome*							
Any recanalization therapy	592	90.38	193	90.61	399	90.27	0.8904
PTCA	524	80.00	174	81.89	350	79.19	0.4528
CABG	74	11.3	22	10.33	52	11.76	0.5865
LVEF							0.0243
> 50%	357	54.75	133	62.44	224	51.03	
41–50%	122	18.71	40	18.78	82	18.68	
31–40%	95	14.57	22	10.33	73	16.63	
≤ 30%	45	6.90	12	5.63	33	7.52	
Undetermined	33	5.06	6	2.82	27	6.15	
Stenting	367	78.92	129	81.13	238	77.78	0.4002
Number of stents							
1	184	50.27	57	44.19	127	53.59	0.3322
2	114	31.15	44	34.11	70	29.54	
3	41	11.20	18	13.95	23	9.70	
> 3	27	7.38	10	7.75	17	7.17	
Any in-hospital complication*	98	14.96	26	12.21	72	16.29	0.1700

*DEC* diagonal ear lobe crease, *HISIHD* history of ischemic heart disease, *COPD* chronic obstructive pulmonary disease, *PAOD* peripheral arterial occlusive disease, *STEMI* ST-elevation myocardial infarction, *NSTEMI* non ST-elevation myocardial infarction, *PCI* percutaneous coronary intervention, *CABG* coronary artery bypass graft, *LVEF* left ventricular ejection fraction

^*^Shock, stroke, bleading, brady/tachycardia, atrial fibrillation, lung edema

DEC 2/3 was significantly more often detected in men (*p* < 0.0001). Also, higher age, hypertension, smoking, hyperlipidemia, history of ischemic heart disease, heart and renal failure, and higher levels of c-reactive protein were significantly associated the presence of DEC. Patients with DEC 2/3 had more often a reduced LV ejection fraction after PCI (*p* = 0.0243) and had a higher 1 year mortality (*p* < 0.0031). STEMI was more often seen with DEC 2/3, whereas NSTEMI with DEC 1/2 (*p* = 0.0049).

No frequency differences were seen between right and left ear (see Table 2). A total of 442 (67.5%) patients showed DEC grade 2/3 and 150 (22.9%) patients DEC grade 3, whereas 213 (32.5%) presented with DEC grade 0/1.

**Table 2:** Frequency of different diagonal ear lobe crease severity grades (n = 655)

	Severity grade	n	%
Right ear (n = 642)	0	102	15.89
	1	172	26.79
	2	254	39.56
	3	114	17.76
Left ear (n = 640)	0	101	15.78
	1	169	26.41
	2	261	40.78
	3	109	17.03
Ear with strongest severity	0	68	10.38
	1	145	22.14
	2	292	44.58
	3	150	22.90
Aggregate of both ears (n = 627)	0	62	9.89
	1	49	7.81
	2	105	16.75
	3	105	16.75
	4	170	27.11
	5	63	10.05
	6	73	11.64
Grade 2 or 3 on ≥ 1 ear			
No		213	32.52
Yes		442	67.48
Grade 3 on ≥ 1 ear			
No		505	77.10
Yes		150	22.90

**Fig. 3:**
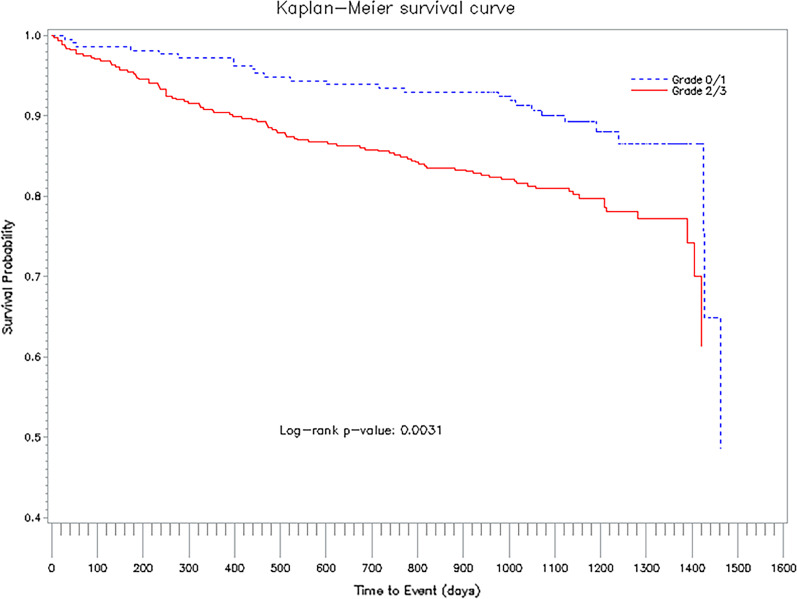
Three-year survival of 655 patients with myocardial infarction according to diagonal ear lobe crease grading

In the unadjusted Cox regression model patients with DEC grade 2/3 had a 1.91-fold higher risk of death (95% confidence interval (CI) 1.23–2.96, *p* = 0.0037) within a median follow-up time of 3 years compared to patients with DEC grade 0/1 (see Fig. 3). After adjusting for age and sex, the hazard ratio decreased to 1.30 (95% CI 0.82–2.04, *p* = 0.2613) (see Additional file 2).

In the fully adjusted Cox regression model, patients with DEC grade 2/3 had a 1.48-fold increased risk of death compared to the DEC Grade 0/1 patient group (CI 0.94–2.34, *p* = 0.0897) (see Table 3). Furthermore, patients with higher age, with left ventricular ejection fraction < 50, history of peripheral arterial occlusive disease and higher levels of c-reactive protein had a significantly higher hazard of death compared with those without these conditions.

**Table 3:** Cox proportional hazards models: associations with all cause 1-year and 3-year mortality in patients with acute myocardial infarction (n = 651)

Variable	Reference	1-year mortality			3-year mortality		
		Hazard ratio	95% CI	*p* value	Hazard ratio	95% CI	*p* value
DEC (grade 2/3)	Grade 0/1	2.57	1.07–6.17	0.0347	1.48	0.94–2.34	0.0897
Gender (female)	Male	0.60	0.31–1.16	0.1301	0.71	0.47–1.08	0.1046
Age [years]		1.05	1.01–1.09	0.0242	1.03	1.01–1.06	0.0057
Diabetes mellitus (yes)	No	1.02	0.54–1.91	0.9632	1.05	0.70–1.56	0.8177
PAOD (yes)	No	1.90	0.98–3.67	0.0566	2.11	1.37–3.25	0.0007
History of stroke (yes)	No	1.56	0.75–3.23	0.2334	1.55	0.96–2.49	0.0717
Chronic kidney disease (yes)	No	1.19	0.57–2.46	0.6423	1.39	0.88–2.22	0.1628
Reinfarction (yes)	No	1.39	0.69 –2.83	0.3595	0.83	0.55–1.25	0.3784
Any recanalization therapy (yes)	No	0.34	0.15–0.76	0.0088	0.63	0.37–1.10	0.1046
LVEF < 50	≥ 50	2.27	1.16–4.42	0.0161	2.21	1.47–3.32	0.0002
1-VD	3-VD	0.64	0.25–1.65	0.3531	0.74	0.42–1.31	0.2965
2-VD	3-VD	1.28	0.64–2.58	0.4839	1.21	0.77–1.91	0.4107
VD: Others, missing	3-VD	1.12	0.38–3.23	0.8415	1.39	0.67–2.87	0.3735
CRP > 3 mg/dl	≤ 3 mg/l	4.02	2.18–7.41	< 0.0001	3.13	2.09–4.68	< 0.0001
CRP data missing	≤ 3 mg/l	1.29	0.38–4.40	0.6861	0.94	0.42–2.08	0.8768

*CI* confidence interval, *DEC* diagonal ear lobe crease, *PAOD* peripheral arterial occlusive disease, *LVEF* left ventricular ejection fraction, *VD* vessel disease, *CRP* c-reactive protein

In addition, an analysis restricted to the first year after AMI was performed. During the first year, 6 patients (2.8%) with DEC grade 0/1 and 42 patients (9.5%) with grade 2/3 died. In the unadjusted Cox regression model patients with DEC grade 2/3 had a 3.52-fold higher risk of death (95% CI 1.50–8.28, *p* = 0.0039) compared to patients with DEC grade 0/1. After adjusting for age and sex, the hazard ratio decreased to 2.14 (95% CI 0.89–5.14, *p* = 0.0879). In the fully adjusted Cox regression model, patients with DEC grade 2/3 had a 2.57-fold increased risk of death compared to the patients with DEC grade 0/1 (CI 1.07–6.17, *p* = 0.0347) (see Table 3).

Sensitivity analyses using other categorizations of DEC (grade 3 of at least one ear, highest grade of both ears, sum of grades of both ears) overall confirmed these results (see Additional file 2). Unadjusted hazard ratios ranged between 1.13 (95% CI 1.02–1.26, *p* = 0.0201) for the sum of grades of both ears and 1.97 (95% CI 0.98–3.97, *p* = 0.0585) for the highest grade of both ears (3 vs. 0). After adjusting for other covariables, the hazard ratios decreased and were not statistically significant.

## Discussion

The analysis of 655 patients with AMI showed that presence and extent of DEC were positively associated with mortality 3 years after AMI. After adjusting for further potential confounding variables, patients with DEC 2/3 had a 1.48-fold increased risk of death compared with patients with DEC 0/1, but this finding failed statistical significance. However, in the first year after AMI the association between DEC and mortality was considerably stronger and also statistically significant.

An association between DEC and CAD was already assumed in 1973 by the first describer Frank [4]. Frank himself proposed in 1977 that this sign should be considered in addition to classic risk factors [12]. Hereafter, numerous studies have confirmed a higher risk of CAD in persons with DEC, while others failed to find an association [13, 14]. Of importance due to its impressive number of 10.885 individuals is the Copenhagen City Heart Study. During the 35 years of follow-up DEC was associated with increased risk of ischemic heart disease and myocardial infarction independent of age and other cardiovascular risk factors [15]. A meta-analysis by Lucenteforte from 2014 summarized the available data and strongly supported the hypothesis that DEC could be a marker for CAD. Based on 37 studies in 31.188 participants, an overall pooled sensitivity of 0.62 (95% CI 0.56–0.67), a specificity of 0.67 (95% CI 0.61–0.73) and an OR of 3.27 (95% CI 2.47–4.32) was found [14].

Beyond the investigations on the association between DEC and risk of CAD, only a few studies have addressed the prognostic value of DEC. On autopsy of 520 forensic cases DEC was observed in 55% and strongly correlated with CAD in both genders. The sensitivity of DEC was 75% and the positive predictive value was 68%. Interestingly, individuals under the age of 40 years had the highest positive predictive value of 80%. The authors also reported a correlation of DEC with sudden cardiac death in men [16]. Within patients referred for coronary angiogram, a Chinese study revealed that at presence of more than four risk factors and of bilateral DEC a higher chance of major adverse cardiac events (MACE) after successful percutaneous coronary intervention (PCI) [17]. Elliott et al. [18] also followed patients referred for coronary angiography. After adjustment for ten established cardiac risk factors, the relative risk for future cardiac events was significantly elevated with DEC (1.33 for unilateral, and 1.77 for bilateral DEC compared to no DEC) [18].

In the present study, 89.6% of the AMI patients had any manifestation of DEC. This finding is consistent with studies that demonstrated a higher risk of CAD in persons with DEC. Moreover, the present study confirmed that DEC is strongly related with age, but the higher mortality risk associated with DEC remains irrespective of the chronological age. Although the pathophysiological mechanism of the association between DEC and occurrence of CAD and the survival after AMI is still unclear, there are a few suggestions from other studies. It was hypothesized that DEC is a simple indicator for accelerated biological ageing since DEC is rare in infants [19] and shortened telomeres in peripheral white blood cells were found in patients with DEC [8]. Studies also suggested that DEC might be a manifestation of a generalized vascular disease [20], since DEC was associated with brachial-ankle pulse wave velocity and aortic intima-media thickness in persons without CAD. Interestingly, Liu et al. [21] found a correlation between the Syntax score as a risk marker regarding complexity of stenosis and DEC suggesting a direct effect on atherosclerosis.

Since DEC can be easily detected by physical examination, the role of this indicator in the assessment of post-AMI risk for adverse outcomes should be investigated in further studies. Previous studies already suggest that DEC in combination with other cardiac risk factors may improve CAD risk assessment [17, 22]. The results of the present study showed that the strength of the association between DEC and mortality attenuates with increasing time after the AMI event. This might be explained by effects of variables which could not be considered in this study, though affecting long-term survival, such as medication or newly diagnosed risk factors. Moreover, in the analysis of 1-year mortality there was only a small number of fatal cases (n = 6) in the group with DEC grade 0/1, which may have distorted the results.

A limitation of our study is the inclusion of patients with residency in Germany and at the age of 25–84 years which reduces transferability of results to other countries and age groups. The small number of fatal cases in the group of patients with DEC grade 0/1 may have limited the validity of the statistical analysis on 1-year survival. In addition, residual confounding cannot be completely ruled out. Strengths are its consecutive enrollment, standardized data acquisition of the registry, double judgement of DEC classification, consideration of relevant covariables, and sensitivity analyses with different classifications of DEC.

## Conclusions

To our knowledge, there are no studies which have investigated the association between DEC and survival in a consecutive AMI population. Depending on the follow-up time, a slightly to moderately increased mortality risk of AMI patients with DEC was observed in the present study. If these effects can be confirmed by larger studies, clinical implications may include prolonged rhythm monitoring, more aggressive risk factor reduction, extended dual platelet inhibition, intensive heart failure management (e.g. early initiation of angiotensin/neprilysin inhibition), and in-patient rehabilitation of AMI patients with DEC.

## Supplementary Information

**Additional file 1.** Angiographic findings within the study population with (grade 2/3) and without (grade 0/1) diagonal earlobe crease (DEC) (n = 655).

**Additional file 2.** Association between diagonal earlobe crease (DEC) and 3-year all cause mortality in patients with AMI (n = 651).

## Data Availability

Data cannot be made available for the public, because the patient consent did not include such an agreement. However, data may be made available for selected research questions and researchers on request to the authors. Please contact Dr. Inge Kirchberger by email: Inge.Kirchberger@med.uni-augsburg.de, or Prof. Christine Meisinger Christine.Meisinger@med.uni-augsburg.de. The Homepage of the Chair of Epidemiology is http://www.uni-augsburg.de/med/epidemiologie.
