# Supplementary material for: Diagonal earlobe crease and long-term survival after myocardial infarction
Source: BMC Cardiovasc Disord. 2021 Dec 16;21:597. doi: 10.1186/s12872-021-02425-4 (PMC8679982; doi:10.1186/s12872-021-02425-4)
Supplement: Supplementary file 1 — Additional file 1. Angiographic findings within the study population with (grade 2/3) and without (grade 0/1) diagonal earlobe crease (DEC) (n = 655). [file 12872_2021_2425_MOESM1_ESM.pdf]

Angiographic findings within the study population with (grade 2/3) and without (grade 0/1) diagonal earlobe crease (DEC) (n=655)

	Total		DEC 0/1		DEC 2/3		p-value
	**n**	**%**	**n**	**%**	**n**	**%**	
**1-VD**	167	26.13	59	27.96	108	25.23	0.1543
**2-VD**	190	29.73	71	33.65	119	27.80	
**3-VD**	265	41.47	76	36.02	189	44.16	
**Main stem affection**	14	2.19	3	1.42	11	2.57	
**LIR / no CAD**	3	0.47	2	0.95	1	0.23	
**Coronary artery dominance**							
**left**	60	12.99	25	15.82	35	11.51	0.3257
**right**	62	13.42	18	11.39	44	14.47	
**codominant**	340	73.59	115	72.78	225	74.01	
**Stent PCI**	367	78.92	129	81.13	238	77.78	0.4002
**Number of stents**							
**1**	184	50.27	57	44.19	127	53.59	0.3322
**2**	114	31.15	44	34.11	70	29.54	
**3**	41	11.20	18	13.95	23	9.70	
**>= 4**	27	7.38	10	7.75	17	7.17	
**% stenosis**							
**Main stem**							
**0**	381	81.76	137	86.16	244	79.48	0.1721
**1 to 50**	47	10.09	15	9.43	32	10.42	
**51-99**	37	7.94	7	4.40	30	9.77	
**100**	1	0.21	0	0	1	0.33	
**LAD**							
**0**	84	18.03	31	19.5	53	17.26	0.5077
**1 to 50**	59	12.66	24	15.09	35	11.40	
**51-99**	237	50.86	74	46.54	163	53.00	
**100**	86	18.45	30	18.87	56	18.24	
**RCA**							
**0**	118	25.38	41	25.95	77	25.08	0.6703
**1 to 50**	47	10.11	14	8.86	33	10.75	
**51-99**	165	35.48	61	38.61	104	33.88	
**100**	135	29.03	42	26.58	93	30.29	
**CFX**							
**0**	153	32.9	54	34.18	99	32.25	0.022
**1 to 50**	53	11.40	27	17.09	26	8.47	
**51-99**	193	41.51	60	37.97	133	43.32	
**100**	66	14.19	17	10.76	49	15.96	

VD: vessel disease; LIR: luminal irregularities; LAD: left anterior descending artery; RCA: right coronary artery; CFX: circumflex artery
