# Supplementary material for: Diagonal earlobe crease and long-term survival after myocardial infarction
Source: BMC Cardiovasc Disord. 2021 Dec 16;21:597. doi: 10.1186/s12872-021-02425-4 (PMC8679982; doi:10.1186/s12872-021-02425-4)
Supplement: Supplementary file 2 — Additional file 2. Association between diagonal earlobe crease (DEC) and 3-year all cause mortality in patients with AMI (n = 651). [file 12872_2021_2425_MOESM2_ESM.pdf]

Association between DEC and 3-year all cause mortality in patients with AMI (n= 651)

| | **Grade 3 or 2 of at least one ear yes/no** | | **Grade 3 of at least one ear yes/no** | | **Highest grade of both ears 0 - 3** | | **Sum of grades of both ears 0 - 6** | |
|---|---|---|---|---|---|---|---|---|
| | **HR (95% CI)** | **p** | **HR (95% CI)** | **p** | **HR (95% CI)** | **p** | **HR (95% CI)** | **p** |
| **Unadjusted** | 1.91 (1.23 – 2.96) | 0.0037 | 1.66 (1.12 – 2.44) | 0.0110 | 1 vs. 0: 0.78 (0.35 – 1.73) | 0.5427 | 1.13 (1.02 – 1.26) | 0.0201 |
| | | | | | 2 vs. 0: 1.46 (0.74 – 2.87) | 0.2754 | | |
| | | | | | 3 vs. 0: 1.97 (0.98 – 3.97) | 0.0585 | | |
| **Adjusted for age and sex** | 1.30 (0.82 – 2.04) | 0.2613 | 1.16 (0.78 – 1.74) | 0.4677 | 1 vs. 0: 0.57 (0.26 – 1.26) | 0.1644 | 0.96 (0.64 – 1.44) | 0.8395 |
| | | | | | 2 vs. 0: 0.85 (0.43 – 1.71) | 0.6543 | | |
| | | | | | 3 vs. 0: 0.91 (0.44 – 1.92) | 0.8238 | | |
| **Adjusted for all covariables*** | 1.48 (0.94 – 2.34) | 0.0897 | 1.42 (0.93 – 2.15) | 0.1013 | 1 vs. 0: 0.95 (0.42 – 2.16) | 0.9032 | 1.07 (0.95 – 1.19) | 0.2649 |
| | | | | | 2 vs. 0: 1.32 (0.65 – 2.68) | 0.4421 | | |
| | | | | | 3 vs. 0: 1.68 (0.79 – 3.58) | 0.1751 | | |

*Age, sex, reinfarction, diabetes, history of stroke, chronic kidney disease, peripheral arterial occlusive disease, any recanalization therapy, c-reactive protein level ≤ 3 vs. > 3 mg/l, left ventricular ejection fraction < 50 vs. ≥ 50, 1-. 2-. 3-vessel disease
